# Coronavirus fear of contagion and compliance with social distancing measures: evidence for a sample of a Spanish university community

**DOI:** 10.7717/peerj.14771

**Published:** 2023-03-01

**Authors:** David Cantarero Prieto, Paloma Lanza-León, Patricia Moreno, Javier Lera, Francisco Gonzalez, Irene González Rodríguez, Carla Blázquez-Fernández

**Affiliations:** 1 Departamento de Economía, Universidad de Cantabria, Santander, Cantabria, Spain; 2 Research Group of Health Economics and Health Services Management, Valdecilla Biomedical Institute Research (IDIVAL), Santander, Cantabria, Spain; 3 Departamento de Economía Aplicada y Métodos Cuantitativos, Universidad de La Rioja, Logroño, La Rioja, Spain; 4 Primary Care, Cantabria Health Service, Santander, Cantabria, Spain

**Keywords:** COVID-19, Social distancing, Compliance, Behavioural change, University, Spain

## Abstract

**Background:**

Social distancing measures have been one of the core pillars of the strategy against COVID-19 in all the countries. This study aims at understanding what motivates behaviours and compliance with social distancing measures among students and workers from a Spanish public university.

**Methods:**

We carry out two logistics models considering two different dependent variables: not maintaining social relation with non-cohabiting people and not to leave home except for emergencies (*n* = 507, sample is formed by students and workers from the University of Cantabria in the North of Spain).

**Results:**

Being very concerned about getting ill suggests higher risk of not maintaining social relation with non-cohabiting people. Getting older increase the probability of not leaving home except for emergencies as happens with those who are very concerned about getting ill. Young people often living with vulnerable older relatives may affect students’ behaviour.

**Conclusions:**

Our findings suggest that compliance with social distancing measures depends on several factors related to age, the number or kind of cohabiting people and level of concern about getting ill. Policies should address all these factors through a multidisciplinary perspective.

## Introduction

The COVID-19 pandemic has burst into the world by surprise and has brought about government intervention in labour and leisure time issues. Following the World Health Organisation Declaration of the global pandemic situation on March 11, 2020 the government declared a state of alert that had six extensions until June 21, 2020 (Gobiernode España, 2020a). A series of social distancing measures were taken to protect the health and safety of citizens, contain the progression of the disease and strengthen the public health system. These measures include cancelling group events, closing schools and commercial activities, and restricting people’s mobility, among others (Gobiernode España, 2020b).

In this regard, a document from the Centers for Disease Control and Prevention regarding pre-pandemic guidance for mitigating the spread of infectious disease, shows the epidemic curves of two different pandemic scenarios: one with no intervention strategies in place and the other, flatter, where there has been intervention (Qualls et al. , 2017).

In cases of close contact, home quarantines (the most extreme form of social distancing) are being implemented, which are being shown in different studies to have negative psychological effects that can be long-lasting (Brooks et al. , 2020). The compliance degree with the measures is related to the infection risk, such as age, health status and work outside the residence place among others, as well as gender, individual preferences and fines (Papanastasiou, Ruffle & Zheng, 2022).

Social interactions are considered a basic human need, analogous to other fundamental needs, such as food or sleep (Bai et al., 2004). Feeling insufficiently connected to others is associated with profound and long-lasting negative consequences on physical and mental health, including increased mortality (Baumeister & Leary, 1995; Achterberg et al., 2020). In other pandemics (Hawkley & Cacioppo, 2010), isolation and quarantine have precipitated anxiety and depression (Hawryluck et al. , 2004; Jeong et al. , 2016; Venkatesh & Edirappuli, 2020) and even stigmatization and rejection by healthcare professionals (Bai et al., 2004). The recent COVID-19 pandemic has created external stressors in many households. Parents must juggle their children studying at home, working remotely or not being able to work at all. Moreover, it should be taken into account the worry of financial consequences or concerns for the families’ health (Jeong et al. , 2016). In some fields, such as education, the virtual space became the new educational environment with a strong impact on the psychological and academic environment (Lozano-Díaz et al., 2020). In the case of the elderly, isolation measures could increase COVID-19 morbidity associated with affective disorders (Hawkley & Cacioppo, 2010), further harming the elderly whose social relations occur only outside the home (Armitage & Nellums, 2020). This may contribute to low mood, boredom, frustration, anxiety due to fear of contagion or lack of clarity in the actions being taken, or media that increase confusion or fear. It is even possible that the effects of such deprivation of social contact may extend beyond the specific period of physical distance and may affect the population for years to come (Achterberg et al., 2020).

In practice, for individuals there are costs and benefits to staying at home, and the policy success to flatten the curve depends on the extent to which individuals comply with the recommendations. Compliance is related to the match between the expectations we have regarding duration and what is advertised (Briscese et al., 2020).

Due to other pandemics, nonpharmaceutical interventions have been established, also known as community mitigation measures, including among others, social distancing measures, that often are the most readily available interventions to help slow transmission of the virus in communities, which is especially important before a pandemic vaccine becomes widely available (Qualls et al. , 2017). Nevertheless, little is known about individual’s willingness to stay at home and comply with the regulations during the COVID-19 pandemic.

In this study we aim to assess, in a university sample, compliance with these social distancing measures and their impact, to understand what motivates behaviours and compliance with the measures. The decree of the state of alarm in Spain has meant for universities (except virtual universities) to switch to an online version of the studies that until now they had attended in person. Mitigating the effects on mental health requires a coordinated effort from the population, government, media, and health professionals.

## Materials & Methods

Three important issues in this section are the data, the empirical strategy and the variables employed in the article. This information is divided into the following subtitles: “Procedure”, “Participants” and “Measurements and statistical analyses”.

### Procedure

The sample is formed by students and workers from the University of Cantabria in the North of Spain. Data was collected through an electronic survey using LimeSurvey software and the respondent information sheet appears on the survey presentation screen. After that first screen, the informed consent form is included. Hence, the informed consent of the participants has been obtained by clicking the “Start survey” button. All methods were carried out in accordance with relevant guidelines and regulations. All experimental protocols were approved by the Ethics Committee of University of Cantabria (November 26, 2020) (Ethical Application Ref: *CE Proyectos 16/2020*). Consent to participate was obtained by all the participants of the study. Additionally, they were informed about the possibility of asking the research assistants in case of doubt. The questionnaire used in this study was anonymous. The survey was conducted in the period from 14/12/2020 to 19/02/2021, in which Cantabria was at a health alert level 3 and in the midst of the third wave (Instituto Cántabro de Estadistica , ICANE).

### Participants

We have studied a sample that corresponds to the university population. 1,417 answers were registered (total responders, after sending e-mails to the full university community). However, some of them were not complete (missing data) and, thus, we have decided not to include them in the study. Finally, 507 observations were taken into consideration.

### Measurements and statistical analyses

Analysing the sample, the vast majority were women (63.51%), students (55.03%) and the mean age was 35 years old. 9.47% of the respondents declared to live alone, while 30.97% indicated to live in a household consisting of four people. In addition, 34.71% reported not to see people who do not live with them, and 10.06% report not to go outside their home, except for emergencies. Also, 70.41% affirmed to be greatly concerned about getting sick with coronavirus. Looking at household income, 17.36% suggested the interval from €2,674.7 to €3,441.9 per month. Moreover, 13.60% of people participating in this survey declare as impossible to pass one hour without seeing the mobile phone.

In this respect, Table 1 shows variables considered in this article, the description, and main descriptive statistics (mean and standard deviation).

**Table 1:** Variables used, coding and main descriptive statistics.

**Variable**		**Coding**	**Mean**	**S.D.**
*Dependent variables*	*Social distancing habits*	1: if the individual reported that does not meet without-cohabitation; 0 otherwise	0.35	0.48
		1: if the individual reported that does not leave home expect for emergencies; 0: otherwise	0.10	0.30
*Socio-demographic factors*	*Female*	1: female; 0: male	0.64	0.48
	*Age*	Aged of the individual (years)	35.01	15.61
	*Student*	1: respondent is a student; 0: otherwise	0.55	0.50
	*Income_1*	1: if the household annual income is less than 7,847 euros; 0 otherwise	0.02	0.14
	*Income_2*	1: if the household annual income is more than 14,536 euros but less than 16,423 euros; 0 otherwise	0.04	0.20
	*Income_3*	1: if the household annual income is more than 26747 euros but less than 34,419 euros; 0: otherwise	0.17	0.38
*Social Isolation factors*	*Alone*	1: if the respondent lives alone; 0: otherwise	0.09	0.29
	*Mobile dependence*	1: if the respondent can be less than an hour without looking at the mobile phone; 0: otherwise	0.14	0.34
*COVID-19 factors*	*COVID-19 concern*	1: if respondent is very worried about getting infected; 0: otherwise	0.70	0.46

**Notes.**

Observations = 1,069. Source: Authors’ elaboration.

The purpose of this article is to analyse the effects of social distancing among students and workers of the University of Cantabria. In pursuit of that, we examine associations between only maintaining social relation with cohabiting people and certain socio-demographics, health, and COVID-19 worries variables. Due to the nature of the potential variables of interest, the proposed methods are the reserved for limited dependent variable. In a first step we analyse which factors affect the probability of not maintaining social relation with non-cohabiting people and in a second step we analyse the probability of not to leave home except for emergencies. More specifically, both cases can be shown here under.

In the first case, we define the dependent variable as:

\begin{eqnarray*}{y}_{i}= \left\{ \begin{array}{@{}l@{}} \displaystyle 1, \text{If the individual reported not to mantain relation with non cohabiting people}\\ \displaystyle 0, \text{Otherwise} \end{array} \right. \end{eqnarray*}

In the second case,

\begin{eqnarray*}{y}_{i}= \left\{ \begin{array}{@{}l@{}} \displaystyle 1, \text{If the individual reported not to leave home except for emergencies}\\ \displaystyle 0, \text{Otherwise} \end{array} \right. \end{eqnarray*}

In both cases, with binary dependent variable and following Stock & Watson (2012) key concept 9.3, one possibility is to use the logit model, which is specified as:

(1) \begin{eqnarray*}\Pr\nolimits \left[ {y}_{i}=1{|}{x}_{1i},{x}_{2i},\ldots ,{x}_{ki} \right] & =F({\beta }_{0}+{\beta }_{1}{x}_{1i}+{\beta }_{2}{x}_{2i}+\ldots +{\beta }_{k}{x}_{ki})\end{eqnarray*}

(2) \begin{eqnarray*}\Pr\nolimits \left[ {y}_{i}=1{|}{x}_{1i},{x}_{2i},\ldots ,{x}_{ki} \right] & = \frac{1}{1+{e}^{-({\beta }_{0}+{\beta }_{1}{x}_{1i}+\ldots +{\beta }_{k}{x}_{ki})}} .\end{eqnarray*}

Discrete choice models are mainly non-linear in parameters and then Ordinary Least Squares is not an adequate estimation method. This problem is solved by using maximum likelihood. The statistical software used in this approximation is Stata 14.

(3) \begin{eqnarray*}LnL \left( \beta {|}{x}_{i} \right) =\sum _{ \left\{ i=1 \right\} }^{n} \left[ \left( 1-{y}_{i} \right) Ln(1-F({x}_{i}^{T}\beta )) \right] +\sum _{ \left\{ i=1 \right\} }^{n}{y}_{i}LnF({x}_{i}^{T}\beta ).\end{eqnarray*}

## Results

In this section, we show the findings obtained when we carry out both logistic regression models. We present our results *via* Odds Ratios (OR) and their associated 95% Confidence Interval (CI). All these findings can be shown in Table 2.

The OR points out the connection between the average odds of reporting not to maintain relation with non-cohabitants at the outcome compared with the average odds when the respondent maintain contact with non-cohabiting people. In this sense, whether we obtain an OR lower than 1, it indicates a reduction in the dependent variable (no contact with non-cohabiting people) when we measure the outcome. By contrast, if we obtain an OR higher than 1, it supposes an increase. Next, we mainly focus on those characteristics which are significant and imply greater probability of not maintaining social relation with non-cohabiting people or not to leave home except for emergencies.

In the first case, as we have previously mentioned, our dependent variable is not maintaining social relation with non-cohabiting people. There is a positive relationship between being older and the probability of not maintaining social relation with non-cohabiting people (OR: 1.194 CI 95% [1.077–1.324]). In addition, being very concerned about getting ill (OR: 2.275 CI 95% [1.408–3.677]) suggests higher risk of not maintaining social relation with non-cohabiting people. The same happens when we are focused on two variables related to household income such as lower than €748.7 per month (OR: 3.931 CI 95% [1.055–14.647]) and the interval between €1,453.6 and €1,642.3 (OR: 2.488 CI 95% [0.958–6.465]).

**Table 2:** Logistic empirical results (odds ratios and 95% confidence intervals).

**Variable**		**Does not meet without-cohabitation**			**Does not leave home expect for emergencies**		
		**OR**	**95% CI**	*p*-value	**OR**	**95% CI**	*p*-value
*Socio-demographic* *factors*	*Female*	0.930	[0.602–1.437]	0.744	0.902	[0.467–1.744]	0.760
	*Age*	1.194	[1.077–1.324]	0.001	1.119	[0.967–1.296]	0.131
	*Age* ^2^	0.998	[0.997–0.999]	0.006	0.999	[0.997–1.001]	0.211
	*Student*	0.971	[0.526–1.794]	0.927	2.293	[0.913–5.759]	0.077
	*Income_1*	3.931	[1.055–14.647]	0.041	3.942	[0.917–16.945]	0.065
	*Income_2*	2.488	[0.958–6.465]	0.061	1.131	[0.239–5.347]	0.876
	*Income_3*	0.738	[0.436–1-251]	0.260	0.426	[0.159–1.142]	0.090
*Social Isolation factors*	*Alone*	0.445	[0.218–0.910]	0.026	0.300	[0.067–1.341]	0.115
	*Mobile dependence*	0.821	[0.821–0.251]	0.519	0.881	[0.352–2.207]	0.788
*COVID-19 factors*	*COVID-19 concern*	2.275	[1.408–3.677]	0.001	3.200	[1.349–7.595]	0.008
	*Constant*	0.007	[0.001–0.067]	0.000	0.003	[0.000–0.086]	0.001

**Notes.**

OR, odds ratios; CI, Confidence Interval.

Observations = 507.

In the second case, looking at not leaving home except for emergencies as dependent variable, students (OR: 2.293 CI 95% [0.913–5.759]) present greater odds, showing larger probability of not leaving home except for emergencies. In addition, getting older (OR: 1.119 CI 95% [0.967–1.296]) increase the probability of not leaving home except for emergencies as happens with the variable related to being very concerned about getting ill (OR: 3.201 CI 95% [1.349–7.595]). Finally, associated with income, individuals who report incomes lower than €748.7 per month (OR: 3.941 CI 95% [0.917–16.945]) and those between €1,453.6 and €1,642.3 (OR: 1.131 CI 95% [0.239–5.347]) show larger probability of not leaving home except for emergencies.

In both estimations, the pseudo R2 is around the 11% and the model is statistically significant because the Prob >chi2 is less than 0.05 suggesting good model fit and specification. Moreover, the overall rate of correct classification is estimated to be 71.60% in the first estimation (“not maintaining social relation with non-cohabiting people”), with 83.99% of the normal weight group correctly classified (specificity) and only 48.30% of the low weight group correctly classified (sensitivity). For the second estimated model, (“not leaving home except for emergencies”) the estimated overall rate of correct classification is still higher, concretely, 83.94%.

## Discussion

The distancing measures since the beginning of the COVID-19 pandemic have had a strong impact on society which, in turn, have had effects on the population’ mental and physical health (Gasteiger et al. , 2021).

Noncompliance is harmful both for the noncompliant and for the community; as such, there are additional cost-benefit considerations, and potentially psychological mechanism, at play. Early application of social distancing measures has been associated with an important reduction in COVID-19 mortality (Piovani et al. , 2021).

This analysis makes it possible to approximate the most relevant consequences on the University of Cantabria workers and students. To do so, we have analysed two behaviours: not meeting with non-cohabitants and not leaving home except for emergencies.

Firstly, the fact of not meeting with non-cohabitants is positively associated with age and the fear towards COVID-19. Our results are consistent with previous literature which demonstrates the elder’s especial vulnerability to COVID-19 (Lithander et al. , 2020). Regarding gender, women in our study comply equally than men. In other studies, women comply with the measures somewhat more than men. Perhaps it is due to the high percentage of women in our sample (Franzen & Wöhner, 2021).

Secondly, we show similar results for students and the workers at the University of Cantabria regarding the question of not leaving home except for emergencies. These two groups are more prone to stay at home unless an emergency takes place. On the one hand, young people usually live with older relatives who are more vulnerable to the COVID-19 effects. This fact may affect students’ behaviour and make them be more cautious in their social contacts. As previous literature demonstrated, young people are very concerned of pandemic’s impact on others (Larcher et al. , 2020). On the other hand, elderly workers are more vulnerable to COVID-19 which explains their behaviour of staying at home (Lithander et al. , 2020). Our results are similar to the ones found by Coroiu et al. (2020). These authors conclude the existence of some compliance facilitators such as self-protection desire, feeling a responsibility to protect other, having the possibility of working/study remotely.

It is important to remark that our sample only covers a public university which is a very specific framework and may drop different results when comparing to the ones related to society in general. For example, nearly 100% of respondents in the low socioeconomic group belong to the student’s group. This is the reason why our results are not consistent with previous research demonstrating that low socioeconomic individuals are not able to reduce their mobility and social contact as their higher socioeconomic peers (Chang et al., 2021).

The lack of socialization and personal relationships, possibly substituted by a greater use of technological media (mobile phones, among others), has been studied through binary variables that include the frequency with which the individuals included in the study stay with non-cohabitants, go to a bar or restaurant, and leave the house during the weekends for different reasons.

Nevertheless, we would like to point out the existence of some limitations. Firstly, the responses to the survey may have been influenced by the so-called goal-gradient effect hypothesis which posits that motivation to reach a goal increases monotonically with proximity to the desired end state (Bonezzi, Brendl & De Angelis, 2011), as the survey coincided with the start of the COVID-19 vaccination on December 27, 2020 (Gobiernode España, 2021). Secondly, a limitation of our empirical model is that the likelihood of compliance may depend on many factors, not only age, sex, health status, possibility of working from home, but also the number of cases at the regional level, political views, social capital, threat of a fine, possibility of income subsidy, confidence in public institutions, attitude towards risk, risk preferences or the enforceability of the regulations between others (Papanastasiou, Ruffle & Zheng, 2022; Gualda et al. , 2021; van Osch, van den Hout & Stiggelbout, 2006; Painter & Qiu, 2020). Thirdly, we have not studied the social desirability bias either, resulting from the desire of respondents to avoid embarrassment and project a favourable image to others (Fisher, 1993). Moreover, we just include a sample from the university environment which may not the Spanish population as a whole.

Further research should address the increase of social rejection of elderly people (ageism) or the growth of individualistic behaviors or the long-term psychological effects that remain in societies long after the pandemic has ended (Sikali, 2020).

## Conclusions

The COVID-19 pandemic entailed several restrictive measures of social distancing to reduce the probability of getting ill. Our study tried to disentangle how the personal and socioeconomic characteristics have affected university students and workers’ behaviour related to the compliance of social distancing measures.

The success of the policies on “flattening the curve” (Boumans, 2021) depends on the extent in which individuals are willing to comply with the directives. However, little is known about individuals’ willingness to stay at home and comply with these regulations during the pandemic. We demonstrate that age, the fear of contagion and the socioeconomic gradient have played a key role in determining the level of compliance with social distancing measures. According to our results, we strongly recommend the following: first, tackling socioeconomic inequalities that increased dangerous behaviours, monitoring the long-term effects of the pandemic, evaluating the increase of ageism.

It is important across different countries to document important drivers of compliance with social distancing, understanding who chooses to practice (or not) social distancing—and why—is crucial for the design of effective public service campaigns, both now, and during the occurrence of future pandemics. Thus, policymakers should focus on the people targeted on such campaigns and what specific beliefs must be addressed.

##  Supplemental Information

10.7717/peerj.14771/supp-1 Supplemental Information 1 Raw data

10.7717/peerj.14771/supp-2 Supplemental Information 2 Survey change in habits due to COVID19 UC: English version

10.7717/peerj.14771/supp-3 Supplemental Information 3 Survey change in habits due to COVID19 UC: Original version

## Additional Information and Declarations

### Data Availability

The following information was supplied regarding data availability: The survey used to collect the data and the results are available in the Supplementary Files.
